# Paraneoplastic neurological syndrome associated with a pulmonary carcinoid tumor: a case report

**DOI:** 10.1093/jscr/rjaf559

**Published:** 2025-08-29

**Authors:** Yeni Arroyave Guerrero, Luis Gerardo García-Herreros Hellal, Julio Cesar Granada Camacho

**Affiliations:** Thoracic Surgery Fellow, Universidad El Bosque, 9th Avenue No. 131A–02, Bogotá 110111, Colombia; Thoracic Surgery Department, National Cancer Institute, 1st Street No. 9–85, Bogotá 110321, Colombia; Thoracic Surgery Department, Fundación Santa Fé de Bogotá, 119th Street No. 7–75, Bogotá 110111, Colombia; Thoracic Surgery Department, Fundación Santa Fé de Bogotá, 119th Street No. 7–75, Bogotá 110111, Colombia

**Keywords:** paraneoplastic syndromes, nervous system, carcinoid tumor, neuroendocrine tumors, antibodies, case reports

## Abstract

Paraneoplastic neurological syndromes (PNS) are a rare and heterogeneous group of immune-mediated neurological disorders. We report the case of a 32-year-old woman with progressive and severely disabling neurological symptoms. After excluding alternative causes, she was diagnosed with an atypical pulmonary carcinoid tumor. Surgical resection led to partial clinical improvement. This case underscores the importance of considering PNS in patients with unexplained neurological symptoms and highlights the need to investigate for underlying neoplasms as a potential trigger.

## Introduction

Pulmonary carcinoids are neuroendocrine tumors (NETs) accounting for 2% of primary lung tumors with ~10% being functional [1, 2]. Paraneoplastic neurological syndromes (PNS) are a rare and heterogeneous group of immune-mediated neurological disorders; Only sporadic cases of pulmonary carcinoids associated with PNS have been reported [3–5]. We present a case of a functional pulmonary NET with symptoms suggestive of PNS, following The CAse REport (CARE) guidelines [6].

## Case report

A 32-year-old woman with hypothyroidism, depression in remission, Raynaud’s phenomenon, positive antinuclear antibodies (ANA) (1:320, homogeneous pattern), and a nailfold capillaroscopy suggestive of early systemic sclerosis, presented with a 7-month history of numbness, paresthesias, pain, and progressive weakness, initially in the upper limbs, later involving the lower limbs. These symptoms limited daily activities such as combing her hair, feeding herself, holding objects, and climbing stairs.

Physical examination revealed a shallow thoracic breathing pattern without signs of respiratory distress, puffy hands, Raynaud’s phenomenon, and bilateral palmar telangiectasias. Muscle strength was decreased in all muscle groups, predominantly proximally, including facial, cervical, and limb muscles. Generalized allodynia was also present, particularly in the thoracic region.

**Figure 1:**
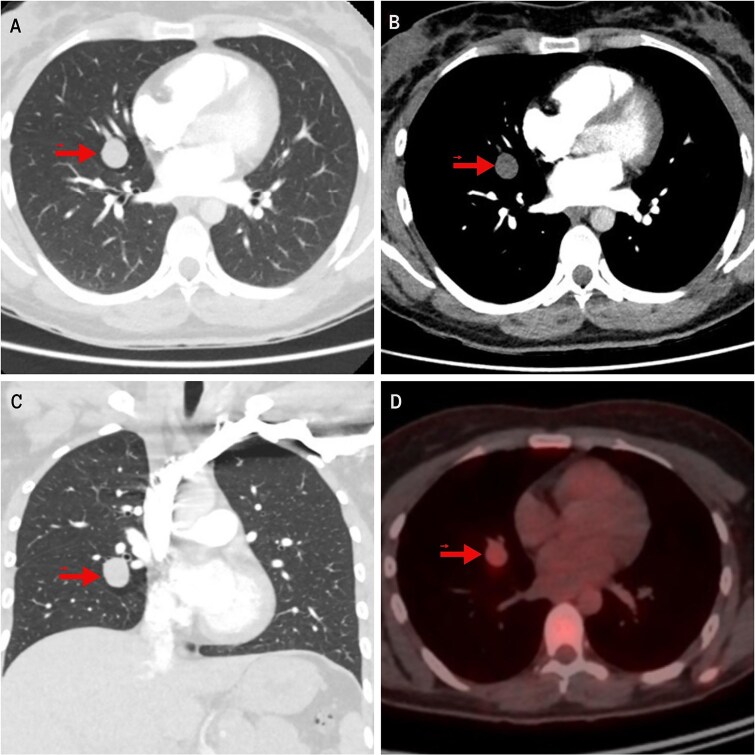
Imaging findings of the pulmonary carcinoid tumor. Contrast-enhanced chest CT. (A, B) Axial views (lung and mediastinal windows); (C) Coronal view (lung window); (D) FDG-PET scan. The arrow indicates a well-circumscribed, round lesion measuring 16 × 19 × 17 mm, with soft tissue density, centrally located in the medial segment of the right middle lobe, and showing mild hypermetabolism on image (D).

In the evaluation of her dyspnea, a chest computed tomography (CT) angiography was performed, ruling out pulmonary embolism but detecting a homogeneous, well-circumscribed pulmonary nodule measuring 19 mm in the middle lobe (ML). On fluorodeoxyglucose positron emission tomography (FDG-PET), the lesion showed mild hypermetabolism with a standardized uptake value (SUV) of 2.8 (Fig. 1). A low-grade NET was suspected. Two transbronchial biopsies were inconclusive. Serum chromogranin A and 24-hour urinary 5-hydroxyindoleacetic acid (5-HIAA) levels were elevated, indicating a functional NET.

Autoimmune workup discarded systemic sclerosis and inflammatory myopathy; magnetic resonance imaging (MRI) of the pelvic and shoulder girdles was normal. The patient reported sicca symptoms. ANA remained positive (AC-4 pattern), but negative anti-Ro antibodies did not support Sjögren’s syndrome.

Nerve conduction studies, limb electromyography, autonomic testing, and cerebrospinal fluid (CSF) analysis were normal, as were brain and spinal MRIs. Although the criteria for a defined neurological disorder were not met, her clinical manifestations progressed to severe functional impairment, including inability to walk or maintain head and trunk posture. A PNS was suspected despite a negative serum panel for paraneoplastic antibodies such as NMDA, AMPA, GABA, mGluR, DPPX, IgLON5, LGI1, CASPR2.

**Figure 2:**
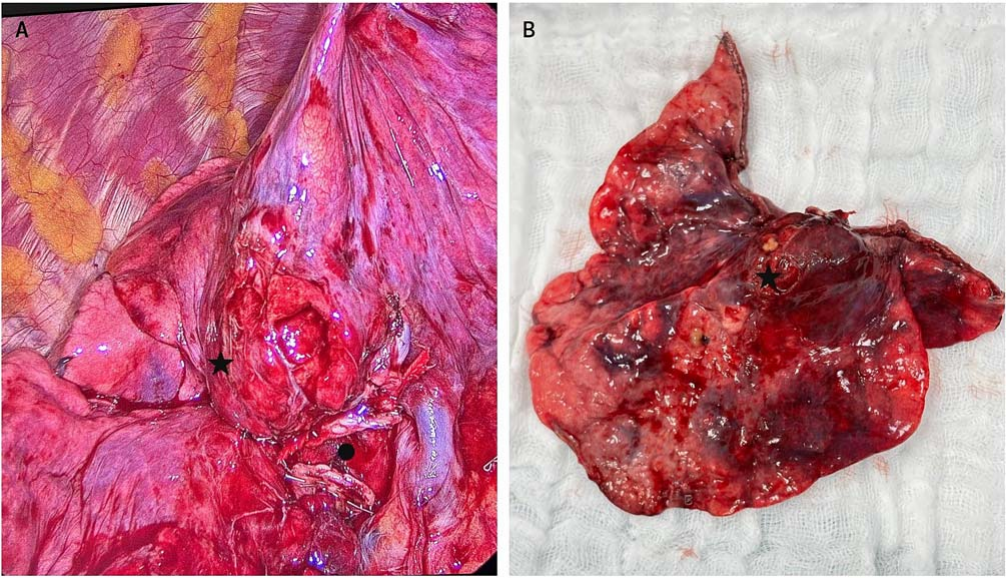
Intraoperative view of the atypical carcinoid tumor in the middle lobe. (A) Marked with ● middle lobe hilum transected with a stapler; to its right, part of the intact hilum of the right upper lobe. (A, B) Marked with ★ carcinoid tumor.

About 28 days after admission, the patient underwent a video-assisted thoracoscopic middle lobectomy with mediastinal lymph node dissection; intraoperative findings included pleural adhesions, inflammatory changes in the hilum, and a 2 cm centrally located ML lesion (Fig. 2). Postoperatively, she remained 48 hours in the intensive care unit (ICU) without requiring mechanical ventilation. By postoperative Day 3, muscle strength and respiratory function had improved significantly; she could walk short distances with assistance and showed better swallowing and mastication. Limb allodynia decreased, although thoracic pain persisted and required a paravertebral block. Histopathology confirmed an atypical carcinoid without nodal involvement. She was transferred to another institution eight days later, and further follow-up was not possible.

**Figure 3:**
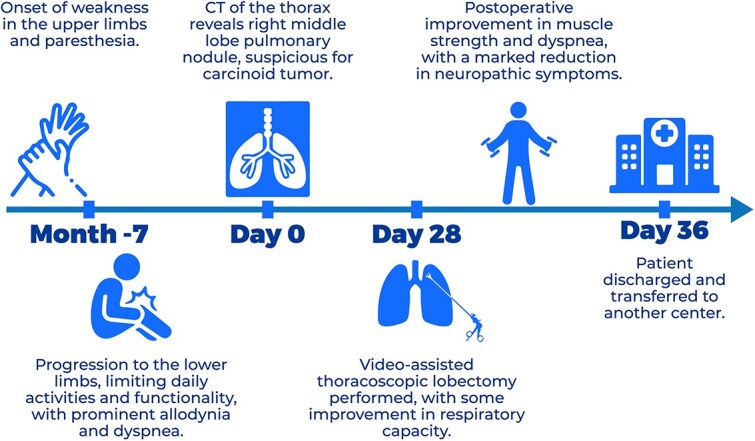
Clinical timeline of events.

The patient’s clinical course is summarized in the timeline (Fig. 3).

## Discusion

PNS are rare, occur in ~1% of cancer patients, most commonly in small cell lung cancer, gynecologic tumors, and lymphomas [3, 7]. This syndrome results from immune-mediated inflammation due to cross-reactivity of antineuronal antibodies against shared tumor and neural antigens; antibodies like anti-Hu, anti-Yo, anti-Ri, anti-Tr, anti-Ma, and anti-SOX1. These can be detected in serum or CSF, although up to 40% of cases may be seronegative [4–8].

There are no absolutely pathognomonic neurological presentations for PNS; however, there are certain high-risk phenotypes that frequently have a neoplastic etiology and are often severely disabling. These phenotypes include Lambert-Eaton myasthenic syndrome (LEMS), encephalomyelitis, limbic encephalitis, rapidly progressive cerebellar syndrome, opsoclonus-myoclonus syndrome, sensory neuronopathy (SNN), and enteric neuropathy [2, 7, 8].

The phenotype most consistent with our case is SNN, characterized by an acute or subacute onset of pain, paresthesias, and asymmetric sensory loss—typically starting in the upper limbs—and progressing to significant functional disability involving limbs, thorax, and cranial nerves. Motor neuron involvement with severe weakness and autonomic features may also occur, further supporting a paraneoplastic etiology. In such cases, the term ‘SNN with motor involvement’ is preferred. Over 80% of paraneoplastic SNN cases are linked to small cell lung cancer with anti-Hu antibodies [7, 9–11].

For the diagnosis of PNS, Graus *et al.*, have proposed three levels of diagnostic certainty for PNS: possible, probable, and definite, based on a scoring system—the PNS-Care Score—which considers the type of clinical phenotype, the presence or absence of neuronal antibodies, and the presence or absence of cancer (Table 1) [7].

**Table 1:** PNS-Care score (7).

	**Points**
**Clinical level**	
High-risk phenotype	3
Intermediate-risk phenotype	2
Phenotype not epidemiologically associated with cancer	0
**Laboratory level**	
High-risk antibodies (>70% association with cancer)	3
Intermediate-risk antibodies (30%–70%)	2
Low-risk antibodies (<30%) or negative	0
**Cancer status**	
Cancer found, consistent with phenotype and antibody (if present), or expression of antigens.	4
Cancer not found or not consistent, but follow-up <2 years	1
Cancer not found and follow-up ≥2 years	0
**Score interpretation** ≥8 Definite 6–7 Probable 4–5 Possible ≤3 Unlikely	

This case presented with both sensory and motor manifestations, as well as features suggestive of dysautonomia, including sicca symptoms and dyspnea. We also considered that Raynaud’s phenomenon and palmar telangiectasias could represent manifestations of autonomic dysregulation.

Despite normal electrophysiology and nerve conduction studies, the clinical picture suggested SNN with motor involvement, in the presence of a functional NET; Although no paraneoplastic antibodies were detected in serum, up to 40% of cases may be seronegative, and only intermediate-risk autoantibodies were assessed.

The partial improvement of symptoms after tumor resection highlights the potential therapeutic role of surgery and supports the diagnosis of a PNS, with a probable level of certainty according to the Graus criteria (PNS-Care Score = 7).

Limited reports exist of PNS in association with pulmonary carcinoid tumors. Tschernatsch *et al.* described three: a subacute SNN with anti-Hu antibodies; a limbic encephalitis with tumor expression of neuronal antigen HuD, which may trigger anti-Hu development; and a paraneoplastic myelitis, also with HuD expression [3]. Other reports include a case of LEMS with an atypical carcinoid and another of progressive motor weakness due to a low-grade middle lobe carcinoid—both showing clinical improvement after surgery, although antineuronal antibodies were not assessed [2, 12].

Given the rarity of PNS, treatment options are limited; management relies on tumor resection and immunosuppression to improve neurological symptoms [8, 11].

In conclusion, in patients presenting with neurological features—particularly those fitting high-risk phenotypes and without a clear cause— PNS should be suspected, and a neoplasm ruled out.
